# Grape juice attenuates left ventricular hypertrophy in dyslipidemic mice

**DOI:** 10.1371/journal.pone.0238163

**Published:** 2020-09-03

**Authors:** Ângela Maria Martins, Danielle Aparecida Quintino Silva Sarto, Karine de Paula Caproni, Janaína Silva, Jaqueline Silva, Paulo Sérgio Souza, Leandro dos Santos, Marcos Javier Espino Ureña, Maria das Graças de Souza Carvalho, Brígida Monteiro Vilas Boas, Lidiane Paula Ardisson Miranda, José Antonio Dias Garcia

**Affiliations:** 1 Federal Institute of Education, Science and Technology of the South of Minas Gerais, Machado, Minas Gerais, Brazil; 2 Federal Institute of Education, Science and Technology of the South of Minas Gerais, Muzambinho, Minas Gerais, Brazil; 3 Academic Unity of Serra Talhada, Rural Federal University of Pernambuco, Serra Talhada, Pernambuco, Brazil; 4 Autonomous University of Santo Domingo and Dominican Institute of Agricultural and Forestry Research, Santo Domingo, Dominican Republic; 5 José do Rosário Vellano University, Alfenas, Minas Gerais, Brazil; Medizinische Universitat Graz, AUSTRIA

## Abstract

**Objective:**

We evaluated the effects of grape juice (*Vitis labrusca* L.) on dyslipidemia, resistance to insulin, and left ventricular hypertrophy (LVH) in mice homozygous for the absence of the LDL receptor gene (LDLr -/-) under a hyperlipidemic diet.

**Methodology:**

We divided 30 male mice (3 months old) into three groups (n = 10); the HL group was fed a high-fat diet, the HLU group received a high-fat diet and 2 g/kg/day of grape juice, and the HLS group was fed a high-fat diet and simvastatin (20 mg/kg/day). We assessed the blood pressure profile of the mice. We also determined the levels of C-reactive protein (CRP) and lipid profile, glycemic and insulinemic profiles, and calculated the HOMA-IR. Cardiomyocyte hypertrophy, interstitial collagen deposit, and the expression of CD40 ligand (CD40L) and metalloproteinases 2 and 9 were assessed immunohistologically.

**Results:**

After 60 days, the mice treated with grape juice showed similar results as those of the group treated with simvastatin. The use of grape fruit attenuated dyslipidemia and insulin resistance and significantly increased the levels of high cholesterol density lipoproteins (HDLc). The antioxidant potential of phenolic compounds associated with the increase in HDLc levels in the mice of the HLU group prevented the development of LVH and arterial hypertension since it inhibited the inflammatory response induced by the CD40 pathway and its ligand CD40L. Consequently, there was a lower expression of MMP-2 and MMP-9 and lower serum levels of CRP.

**Conclusion:**

Grape juice has a hypolipidemic and cardiac protective potential, presenting a similar effect as that of simvastatin through a direct antioxidant action of phenolic compounds, or indirectly, via antioxidant action and anti-inflammatory activity of the HDLc. These results suggest that grape juice is a functional food possessing a high potential to prevent cardiovascular diseases.

## Introduction

Cardiovascular diseases (CVD) represent 30% of all causes of death worldwide [1]. Dyslipidemia is a significant risk factor for CVD and its development is directly related to serum levels of low-density lipoprotein cholesterol (LDLc) and triglycerides (TG) and, inversely associated with serum levels of high-density lipoprotein cholesterol (HDLc) [2]. In addition, oxidative stress generated by dyslipidemia has been associated with the appearance of insulin resistance, increased cardiovascular inflammatory markers and left ventricular hypertrophy (LVH) with remodeling involving collagen deposit and expression of metalloproteinases [3,4].

The Inhibitors of hydroxymethyl glutaryl-coenzyme-A reductase (HMG-CoA reductase), better known as statins, are the most validated clinically therapy to reduce cardiovascular events by lowering plasma levels of LDLc, very-low-density lipoprotein cholesterol (VLDLc), inflammatory markers and increase the levels of nitric oxide [5]. However, long-term treatment with statins is associated with muscle complaints, renal and hepatic impairment, hypothyroidism, diabetes, polyneuropathy, and other side effects [6].

The lipid metabolism regulation through diet is an essential target for therapeutic intervention in order to reduce the risk of CVD [7]. The consumption of grape products such as red wine and red grape juice has been shown to have positive effects on human health by increasing the antioxidant capacity [8], reducing the cholesterol levels and proinflammatory markers.

Grapes (*Vitis labrusca* L.) are rich in phenolic compounds and, according to Ramsay (Ramsay et al., 2017), about 46% are anthocyanins. Anthocyanins have great health benefits due to their biological activities, which include antioxidant, anti-inflammatory properties, inhibition of LDLc oxidation, reduction of CVD, and cancer risks [9].

Studies with LDL receptor-deficient mice (LDLr—/ -) showed that when these animals are fed with a hyperlipidic diet, they became susceptible to neointimal lesions [10], with increased arterial oxidative stress with development of atherosclerosis plaque [11]. It also causes LVH with a larger immunoreactive area for CD40L, severe mixed dyslipidemia with reduction of serum levels of HDLc, and insulin resistance [12]. In the present study, the effects of grape juice on dyslipidemia, insulin resistance, and LVH in LDLr -/- mice submitted to a hyperlipidemic diet were evaluated.

## Material and methods

### Grape juice (*Vitis labrusca* L.)

To prepare the juice we used red table grapes from Isabel cultivar (*Vitis labrusca* L.), harvested in the Federal Institute of Education, Science and Technology of South Minas Gerais (IFSULDEMINAS), in the Muzambinho *Campus*. The juice was developed by the sector of Agroindustry Vegetables of the same institution, using a steam drag juice extractor, without the addition of any solute, preservative, and/or water. The juice was kept in a ambar glas vessel under 5°C during all he experiment.

Physicochemical analyzes were carried out in the grape juice to analyze the moisture content, the presence of ashes or mineral material, the amount of total lipids, crude protein, pH and soluble solids. The moisture content was obtained by direct drying in an oven at 105 °C; the percentage of ash was obtained by heating to 550 °C; the amount of crude protein was calculated by the Kjeldahl method adopting the correction factor of 6.25; the pH analysis was performed using pHmeter and the content of soluble solids (°Brix) was obtained by reading a benchtop refractometer with automatic temperature correction. All the analyses were performed according to the methodology described by Pregnolatto and Pascuet (1985) [13].

To analyse the total lipids content we used the Bligh and Dyer techinique [14]. The analysis of total phenolic content was carried out at in the Agricultural Research Company of Minas Gerais (EPAMIG) in Caldas/MG, using the Folin Ciocalteau method with gallic acid as the reference standard [15]. All the analysis were conducted in triplicate.

### Animal protocol

A total of 30 male mice (3 months old), which were homozygous for the absence of the LDL receptor gene (LDLr -/-) generated in background C57BL6 and weighing 22 ± 2g, were studied. The animals were purchased at the Jackson Laboratory (USA) and maintained at the José do Rosário Vellano University (UNIFENAS), Alfenas/MG, with temperature control and light/dark cycle (12h).

The mice were divided into three experimental groups (n = 10); Group HL: The mice received hyperlipid feed with 20% total fat, 1.25% cholesterol, and 0.5% cholic acid; Group HLU: The mice received hyperlipid feed with 20% total fat, 1.25% cholesterol, 0.5% cholic acid, and treated with grape juice at a dosage of 2 g/kg administered via gavage once a day. HLS Group: The mice received hyperlipidemic feed with 20% total fat, 1.25% cholesterol, 0.5% cholic acid and treatment with simvastatin (commercially available) at a dose of 20 mg/kg administered via gavage once a day.

All animals received their respective diets and were treated with water ad libitum. After 60 days of the experiment, all the animals were anaesthetized intraperitoneally using Xylazine/Ketamine (Bayer AS and Parke-Davis^®^), respectively, at the concentration of 10–100 mg/kg. After anaesthesia, blood samples were collected via retro-orbital, using heparinized capillaries, and analyzed for glucose, insulin, C-reactive protein (CRP), TG, total cholesterol (TC) and its fractions.

After the thoracotomy, the left ventricle (LV) was removed and freshly weighed to perform the calculation of ventricular weight (mg) by the animal weight (g) [3]. The experimental procedures were performed in accordance with the guidelines established by the National Council for Animal Experiments Control (CONCEA) and were approved by the Animals Ethics Committee of the Instituto Federal do Sul de Minas Gerais (N°. 03A/2014).

### Resting blood pressure and heart rate measurements

Tail-cuff blood pressure and heart rate were measured in conscious mice before treatment between 10 AM and 12 AM, using a computerized tail-cuff Kent Scientific (XBP 1000) system. The first 6 days of measurements were mostly for training purposes. Data collected during these days were not used for calculations but to assess whether reliable flow waveforms could be obtained in each mouse. On the day of the recordings, sets of 30 measurements were recorded. On average, 20 to 30 blood pressure value measurements were computed for each mouse [16].

### Serum analysis

Serum levels were obtained by blood sample centrifugation and serum glucose concentration was measured by the enzymatic colourimetric method, according to the technique proposed by Trinder [17]. The concentration of insulin was measured using a commercial ELISA (Enzyme-Linked Immunosorbent Assay) kit. The Homa index (HOMAir) to determine the insulin resistance was calculated by the formula: {HOMAir = fasting insulinemia (mU/L) x fasting blood glucose (mmol/L)] /22.5}. For the measurement of serum lipids (TG, TC, and HDLc), an enzymatic assay was used according to Hedrick et al. (2001) [18]. The levels of LDLc were determined by the formula proposed by Friedewald et al. (1972) [19]. The quantitative determination of CRP was done by turbidimetry and photometry (Humastar 300^®^ apparatus) and the results were expressed in mg/dL.

### Morphometric and histological analysis

The left ventricle (LV) was fixed for 48 hours in formalin (10%) and histologically processed according to Junqueira et al. (1979) [20]. The ventricles histological sections were treated with 3% hydrogen peroxide to block endogenous peroxidase activity. The samples were incubated for 12 hours with polyclonal antibody produced in rabbit anti-CD40L (Santa Cruz^®^ 1:50) in a humid chamber. After incubation with the primary antibody, a second incubation with biotinylated secondary antibody (Dako^®^ LSAB kit) was performed for 1 hour at 37 °C. In order to demonstrate the immunoreactive areas, the sections were incubated with peroxidase-conjugated complex (Dako^®^ LSAB kit) for 45 minutes at 37 °C and placed in chromogen solution (50 mg DAB in 50 mL PBS with 3 mL of 10% hydrogen peroxide) for 3 minutes. After counterstaining with Harris hematoxylin (Sigma^®^) for 25 seconds, the blades were mounted and analyzed under an optical microscope and the fractional percentages of the immunoreactive area for LV CD40L were acquired [11].

The ventricles histological sections were stained with Picrosirius red to evaluate and quantify the collagen of cardiac tissue by means of polarized light and with HE (hematoxylin/eosin) for morphometric analysis of cardiomyocytes.

### Protein analysis immunoblotting

LV tissues from mice were pulverized at –80C using a stainless steel pestle and mortar and were resuspended in homogenization buffer of the following composition: 0.1 mM Pipes, 5 mM MgCl2, 5 mM EDTA, 0.5% Triton 3100 (vol/vol), 20% glycerol (vol/vol), and protease inhibitor mixture (Complete, Roche Applied Science). Samples were centrifuged, and the supernatant was collected and assayed for total protein concentration using the Bradford method (BioRad). Equal amounts of protein (200 mg/lane) from 3 left ventricles of each group were resolved by 7.5% (wt/vol) SDS polyacrylamide minigels (Mini-Protean III, Bio-Rad) and transferred onto the nitrocellulose membrane (Amersham Biosciences). The blots were blocked with 5% (wt/vol) nonfat milk (marvel) in buffer containing 10 mM Tris-HCl (pH 7.6), 10 mM NaCl, and Tween 20 (20%, wt/vol) and incubated overnight with rabbit antibodies against CD40L, MMP-2 and MMP-9 antibodies (1:1000 dilutions). Then, appropriate staining controls (GAPDH) were processed. Blots were exposed to horseradish peroxidase–conjugated anti-rabbit IgG secondary antibody (1:2000, Santa Cruz Biotechnology), and immunoreactivity was visualized using an autoradiograph film.11,28 Band intensities were quantified by optical densitometry of the developed autoradiographs.

### Statistical analysis

The obtained data were expressed as the mean ± standard error of the mean (SEM). The analyses of variance (ANOVA), followed by the Tukey test, were used to compare the average between the different groups. The differences were considered significant when p<0.05. All statistical treatments were performed using GraphPad Instat statistical software, version 3.05, for Windows (GraphPad InstatTM, San Diego, CA, USA).

## Results

In the present study, the grape juice presented an average of total phenolic concentration of 1029 ± 24 mg/L. The pH average found was 2.96 and the average of soluble solids was 11.7° Brix. The percentages of moisture, ash, lipids and proteins found were 89.88; 0.16; 0.38 and 0.58, respectively. In the present study, the grape juice presented an average of total phenolic concentration of 1029 ± 24 mg/L. The pH average found was 2.96 and the average of soluble solids was 11.7° Brix. The percentages of moisture, ash, lipids and proteins found were 89.88; 0.16; 0.38 and 0.58, respectively.

In the analysis of the lipid profile, grape juice prevented the increase of TC, LDLc and TG in 42, 46, 31% respectively, in the HLU group, when compared to the HL group. In addition, its use promoted a significant rise in HDLc levels. Similar results were found in the mice in the simvastatin-treated group (HLS) (Table 1). The serum levels of TC and LDLc in the HLS group were lower when compared with the HLU group.

**Table 1:** Comparison of serum levels of lipids, glucose, insulin, Homa index (Homair), CRP and systolic blood pressure (SAP), diastolic blood pressure (DAP), mean arterial pressure (MAP) and LDLr -/- heart rate mice from the HL, HLU and HLS groups.

Group	HL	HLU	HLS
TC (mg/dL)	681 ± 38.2^a^	398 ± 15.0^b^	330 ± 8.0^c^
HDLc (mg/dL)	27 ± 2.7^b^	37 ± 5.2^a^	36 ± 1.9^a^
LDLc (mg/dL)	608 ± 32.0^a^	329 ± 13.3^b^	264 ± 23.1^c^
TG (mg/dL)	230 ± 5.7^a^	159 ± 8.0^b^	158 ± 6.3^b^
Glucose (mMol/L)	5.6 ± 0.2^a^	5.3 ± 0.1^a^	5.3 ± 0.2^a^
Insulin (μg/dL)	6.3 ± 0.6^a^	3.6 ± 0.2^b^	3.2 ± 0.4^b^
Homa_ir_	1.6 ± 0.2^a^	0.8 ± 0.05^b^	0.7 ± 0.06^b^
CRP (mg/dL)	15.2 ± 1.0^a^	6.3 ± 1.5^b^	5.2 ± 1.2^b^
SAP (mmHg)	146±1^a^	140±1^b^	138±1^b^
DAP (mmHg)	130±2^a^	120±3^b^	122±2^b^
MAP (mmHg)	135±3^a^	127±2^b^	128±3^b^
Heart Rate	530±11^a^	527±9^a^	520±3^a^

Values expressed as mean ± standard error of the mean. Different letters indicate significant differences between groups for each variable (p <0.05). Total cholesterol (TC); high density lipoprotein cholesterol (HDLc); low density lipoprotein cholesterol (LDLc); triglycerides (TG) and C-reactive protein (CRP). HL Group: The mice received a high fat diet with 20% total fat, 1.25% cholesterol and 0.5% cholic acid; HLU Group: The mice received a high fat diet with 20% total fat, 1.25% cholesterol, 0.5% cholic acid and treated with grape juice; HLS Group: The mice received a hyperlipidemic diet with 20% total fat, 1.25% cholesterol, 0.5% cholic acid and treatment with simvastatin.

Regarding the glucose levels of the studied groups HL, HLU and HLS do not show statistical differences. However, the animals in the HL group showed insulin and Homair concentration in the blood legs, characterizing insulin resistance. The HLU and HLS groups showed low variations in these parameters when compared with the HL group, however, they did not show statistical differences between them. The concentration of C-reactive protein (CRP) was more pronounced in the HL group, when compared to the HLU and HLS groups, without statistical differences (Table 1).

The present study also demonstrated differences in the blood pressure profile of the animals. The mice in the HL group showed increased systolic blood pressure (SBP), mean arterial pressure (MAP) and diastolic blood pressure (DBP) when compared with the HLU and HLS groups (Table 1).

**Fig 1:**
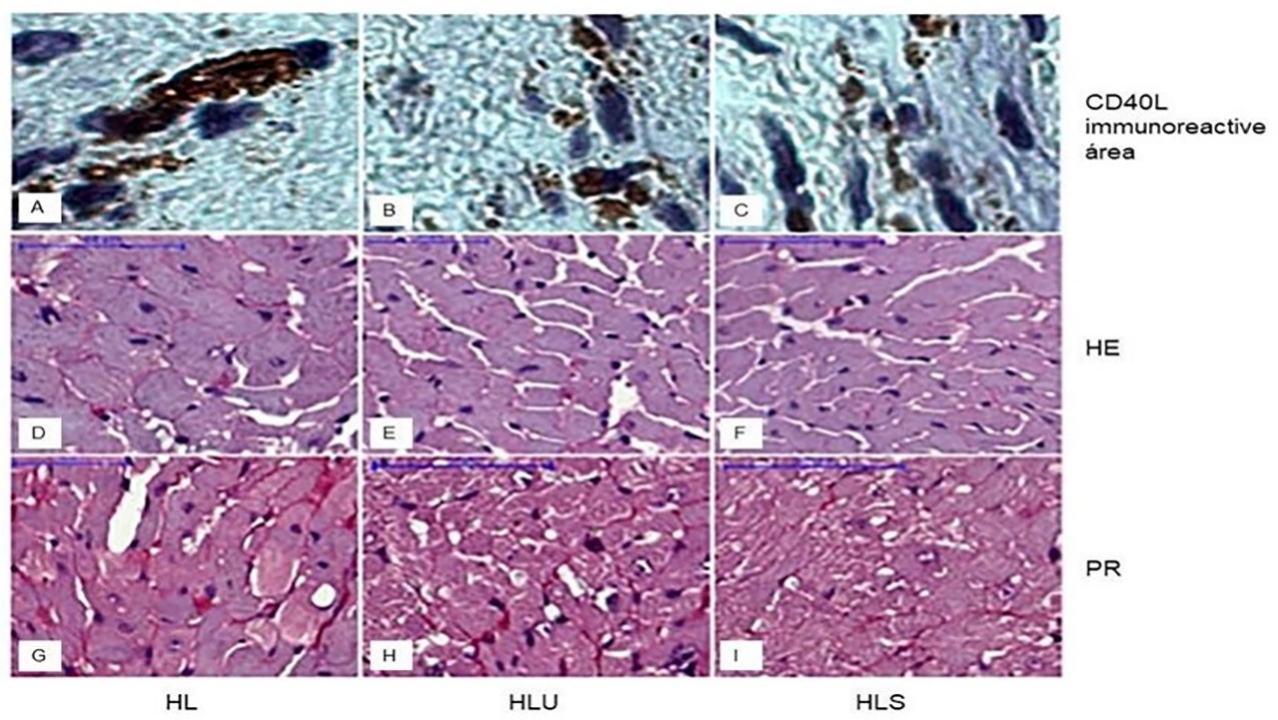
Photomicrograph of transverse histological sections of the left ventricle of mice showing the immunoreactive area for CD40L, cardiomyocyte diameter, and collagen deposition (marked in red). Group HL: received hyperlipid ration; Group HLU: received hyperlipid feed and grape juice; Group HLS: received hyperlipidic ration and simvastatin.

Regarding the weight of the left ventricle (mg) by the weight of the animal (g), the HL group showed an increase in this proportion, followed by an increase in the diameter of the cardiomyocytes, deposition of tissue collagen and immunoreactive area CD40L in the LV when compared to the HLS groups and HLU (Table 2 and Fig 1). The use of grape juice and treatment with simvastatin prevented LVH, demonstrating an anti-inflammatory effect (Table 2 and Fig 1).

**Table 2:** Comparison of left ventricular weight (mg) by animal weight (g), cardiomyocyte diameter (μm), percentage of collagen composition in the LV immunoreactive area (%) and CD40L in the LV (%) in LDLr mice -/- fed a high fat diet; PA group: Received high fat diet; HLU Group: Received high fat food and grape juice; HLS Group: Received high fat food and simvastatin.

	HL	HLU	HLS
Proportion of LV weight (mg)/ animal weight (g)	4.2 ± 0.08^a^	3.6 ± 0.01^b^	3.1 ± 0.09^c^
Cardiomyocyte diameter (μm)	25.0 ± 0.8^a^	22.5 ± 1.0^b^	19.0 ± 1.0^c^
LV Collagen deposition (%)	9.8 ± 1.2^a^	4.2 ± 1.0^b^	4.1 ± 1.1^b^
LV CD40 immunoreactive area (%)	7.0 ± 0.5^a^	3.0 ± 0.3^b^	2.6 ± 1.0^b^

Values expressed as mean ± standard error of the mean. Different letters indicate significant differences between groups for each variable (p <0.05).

**Fig 2:**
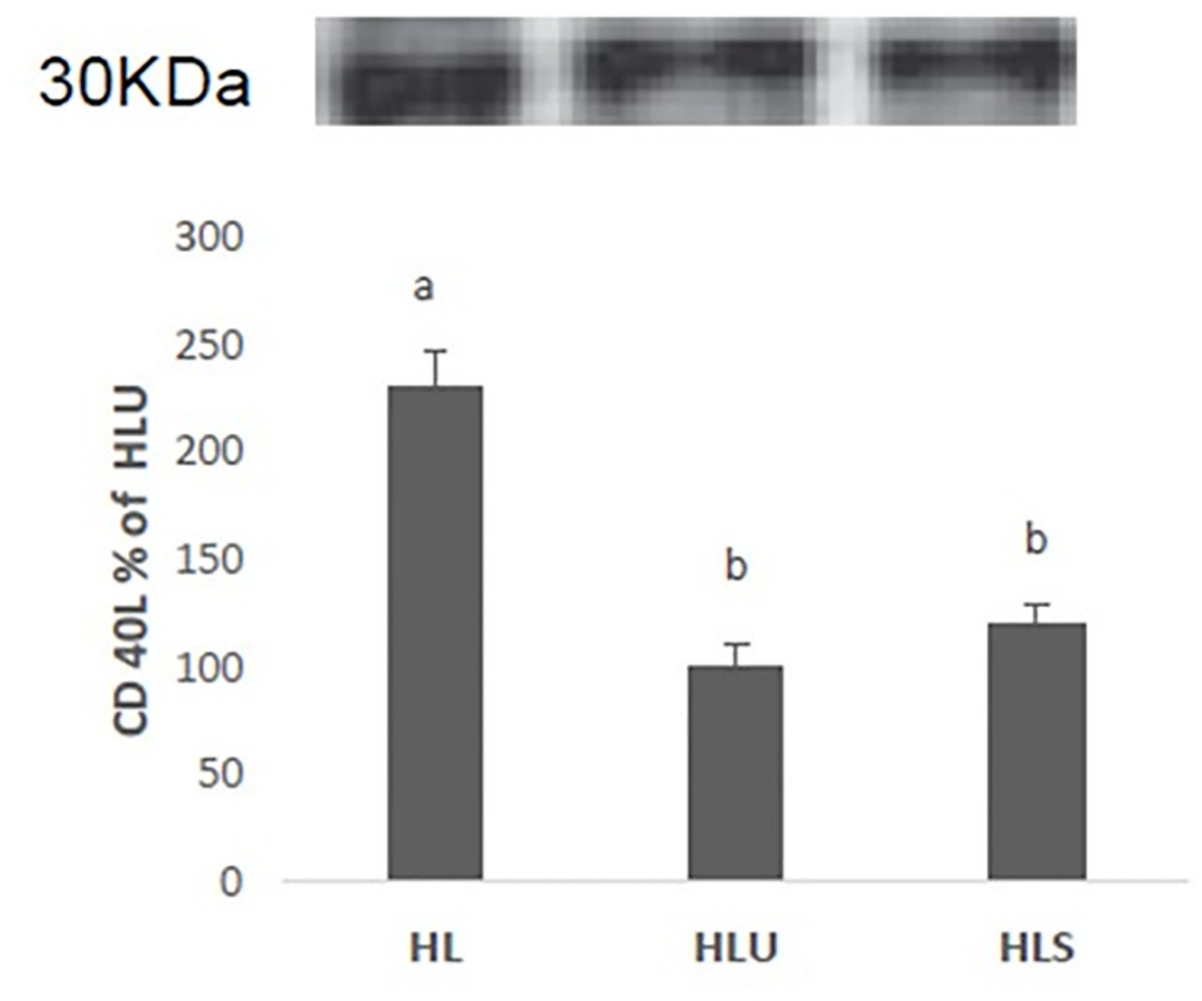
Western Blot analysis of the expression of the CD40 ligand protein (CD40L) LDLr -/- fed a high fat diet (HL), LDLr -/- mice fed a high fat diet and treated with grape juice (HLU), LDLr mice -/- fed a high-fat diet treated with simvastatin (HLS). Values expressed as mean ± standard error of the mean. (ANOVA + Tukey test).

Immunoblotting analyzes showed high expression of the CD40L protein in the left ventricle of mice in the HL group, when compared with the HLU and HLS groups, and there was no difference between these two groups (Fig 2).

**Fig 3:**
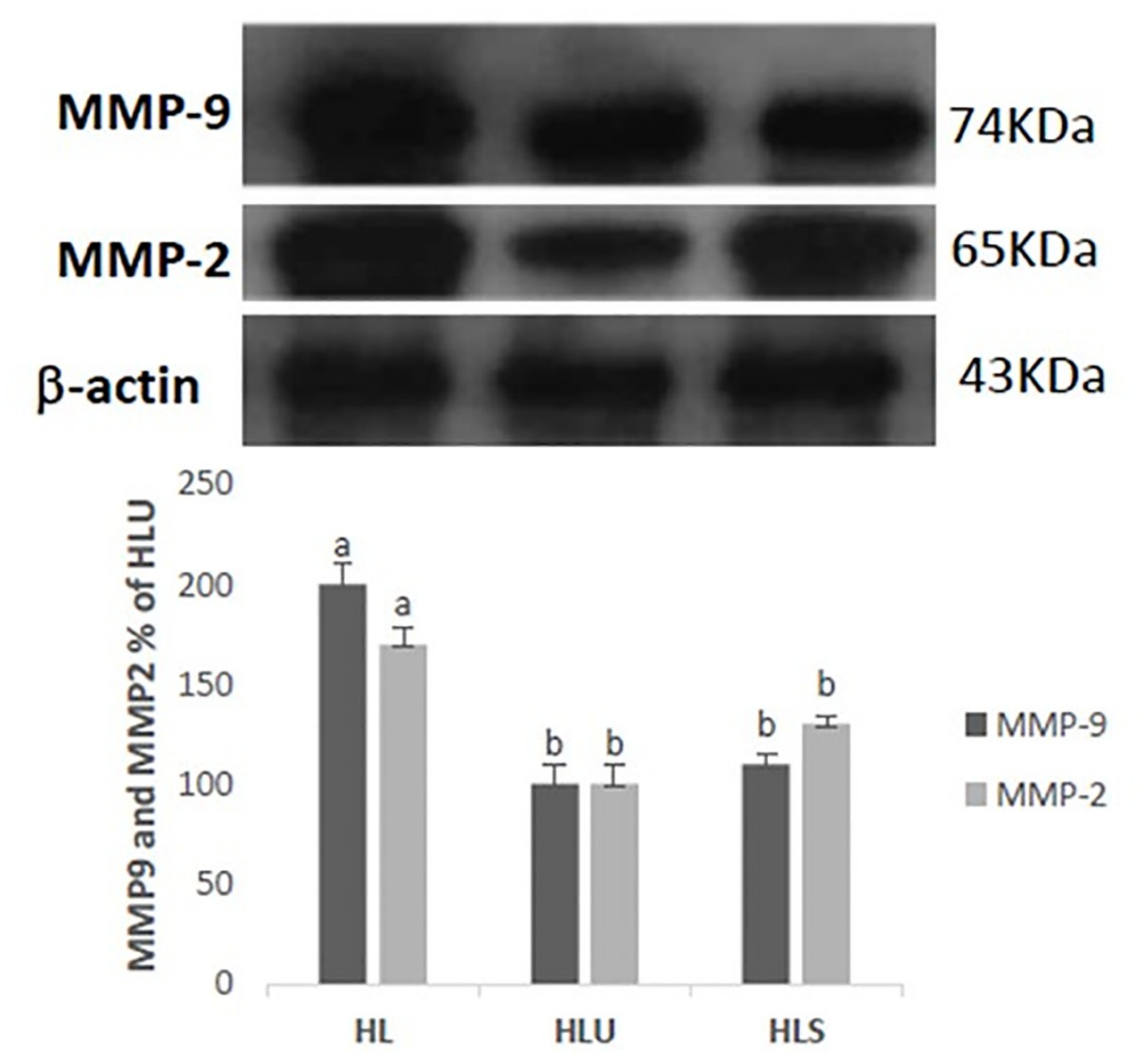
Western Blot analysis of the expression of metalloproteins 2 (MMP-2) and 9 (MMP-9) and β-actin protein in LDLr -/- mice fed a high fat diet (HL), LDLr -/- mice fed with a hyperlipidic diet defined with grape juice (HLU), LDLr -/- mice fed an open hyperlipidic diet with simvastatin (HLS). Values expressed as mean ± standard error of the mean. (ANOVA + Tukey test).

It was observed by immunoblotting analysis that the expression of metalloproteinase 2 (MMP-2) and 9 (MMP-9) showed modulation in their expression in the HLU and HLS groups, showing less expression when compared with the HL group. In addition, the expression of β-actin showed normality in the levels of proteins detected, confirming that the protein loading is the same in the gel (Fig 3).

## Discussion

Studies have indicated that LDLr -/- mice fed a standard diet showed lower arterial oxidative stress [11], higher resistance to the development of arterial lesions [10], and smaller immunoreactive area for CD40L [12] compared to LDLr -/- mice fed a high-fat diet. In the present study, LDLr -/- mice fed a high-fat diet (HL group) exhibited severe mixed dyslipidemia, with reduced HDLc levels, insulin resistance with hyperinsulinemia, LVH, and increased collagen deposit in the extracellular matrix with increased expression of metalloproteinases 2 (MMP-2) and 9 (MMP-9), associated with the activation of the CD40L pathway in the myocardium and arterial hypertension.

CD40L is a transmembrane protein that has a pro-oxidant effect [21]. Interaction with its CD40 receptor induces the production of several potent proinflammatory cytokines, such as IL-1, IL-6, and IL-8 [22], thereby facilitating the development of acute coronary syndrome [21], activation of the NF-kappa β pathway [23], and phosphorylation of KKI (Kappa β kinase inhibitor), which in turn trigger the genes involved in cardiac inflammation and hypertrophy [3]. We observed LVH and an increase in serum levels of CRP, a circulating inflammatory marker, in the mice from the HL group. Furthermore, the activation of the CD40/CD40L pathway in these mice induced an increase in the expression of MMP-2 and MMP-9 in the myocardium, as observed by Horton et al. (2001) in arterial smooth muscle and endothelial cells [24]. Activation of the CD40L pathway also facilitates the uptake of cellular lipids [22], which alter the function and expression of sensitive KATP channels in the myocardium [25], causing cardiac hypertrophy. Hyperinsulinemia is also involved in stimulating LVH in HL mice, including the activation of the ERK/MAPK pathway [26] or the increase of mRNA expression of AT2 receptors [27], as well as the activation of the sympathetic nervous system [28]. Therefore, these effects are, directly or indirectly, related to dyslipidemia, and consequently to the LVH developed in the mice from the HL group.

Metalloproteinases are an essential family of enzymes present in the extracellular matrix and are expressed in cardiovascular cells, such as myocytes, fibroblasts, endothelial cells, smooth muscle cells, and macrophages [29]. Studies suggest that specific MMPs, such as MMP-2 and MMP-9, play a fundamental role in cardiac remodeling and degradation of the extracellular matrix in individuals with dyslipidemia [30,31]. Polyakova et al. (2010) demonstrated that high expression of MMP-2 and MMP-9 is associated with the maturation of collagen in heart failure, indicating an essential role of these enzymes in fibrosis through the activation, deposition, and configuration of collagen. The left ventricular remodeling with collagen deposit observed in the LVH of mice in from the LH group, in the present study, was mediated by an inflammatory process with increased expression of the enzymes MMP-2 and MMP-9, as well as proliferation of cardiac fibroblasts [32].

Grapes are considered to be one of the primary sources of phenolic compounds compared to other fruits and vegetables [33]. The total phenolic content of grape juice varies on average from 400 to 3000 mg/L and depends upon its stage of maturation as well as on the variety of the grape used, geographical origin, type of soil in which they are grown, amount of exposure to sunlight, and juice processing technology [34]. The grape juice used in this study had a similar average of total phenolic content as that found in juices of other varieties of grapes [35,36].

In the present study, treatment with grape juice prevented mixed dyslipidemia and reduced serum levels of HDLc in the mice of the HLU group than of the HL group. Moreover, it hindered insulin resistance, LVH, and ventricular inflammatory process, as indicated by the lower expression of CD40L and lower serum CRP levels. It also led to less collagen deposition in the ventricular tissue followed by lower expression of MMP-2 and MMP-9, and prevented hypertension. These effects are correlated with the content of phenolic compounds present in the grape juice and its antioxidant capacity, thus leading to disease prevention [37].

The hypolipidemic effect of grape juice in the mice of the HLU group may have occurred due to the presence of phenolic compounds, which may have reduced the absorption of cholesterol due to the ability of these compounds to bind to cholesterol, preventing its absorption and causing higher fecal excretion along with bile acids, and other dietary fats [38]. Furthermore, phenolic compounds increased the activity of lipoprotein lipase (LPL), thereby promoting hydrolysis of TG molecules found in lipoprotein particles that resulted in a more significant plasma removal [39]; consequently, reducing TG levels in the HLU group. By contrast, the use of simvastatin in the HLS group prevented the endogenous production of lipids, inhibiting the functioning of the enzyme HMG-CoA reductase [40] to reduce serum lipids levels in this group.

The primary phenol molecules present in grapes are flavonoids (anthocyanins and flavonols), stilbenes (resveratrol), phenolic acids (derived from cinnamic and benzoic acids) and a wide variety of tannins [41]. Several studies have shown that these phenolic compounds can promote health benefits, such as high antioxidant capacity and inhibition of cell proliferation [42,43], in addition to reducing the oxidation of LDLc and platelet aggregation, thus contributing to the reduction of progression of atherosclerotic lesions [44]. In the present study, the phenolic compounds present in grape juice prevented the decrease in serum levels of HDLc and its oxidation, which consequently decreased its hepatic removal [45]. Thus, the use of grape juice in the model studied balanced the relationship between LDLc and HDLc, protecting the heart tissue.

The LDLc/HDLc balance associated with the antioxidant effect of phenolic compounds in grape juice prevented the ventricular inflammatory process, as recorded in the HLU group. These effects may have been due to the antioxidant action of polyphenols (catechin, epicatechin, quercetin, and resveratrol) on reactive oxygen (ROS) and reactive nitrogen (RNA) species [46], thereby decreasing the inflammatory process via a mechanism to reduce the secretion of adhesion molecules (VCAM-1 and ICAM-1). Furthermore, reduction in the production and activity of matrix metalloproteinases and inhibition of CD40-induced JNK and p38 activation may be implicated in beneficial effects of such compounds. Furthermore, grape juice prevented the increased expression of the CD40L protein in the HLU group, which may have prevented an increase in the production of free radicals and expression of proinflammatory cytokines as described in a study by Albers et al. (2004) in individuals with coronary disease [47]. This anti-inflammatory and antioxidant potential demonstrated by the polyphenols present in grape juice were able to prevent LVH in the HLU group, hindering the increase in serum levels of CRP and the inflammatory process with a lower expression of CD40L, MMP-2, and MMP-9. Avellone et al.(2006), who studied healthy men and women, and Estruch et al. (2004) found that grape juice reduced LDL-c and CRP levels and significantly increased HDLc concentrations; these results were similar to those observed in this study [48,49].

A similar cardioprotective potential of grape juice was also observed in the HLS group. In addition to exhibiting hypolipidemic effects, statins decrease oxidative stress [50] by regulating the molecular pathways of NADPH oxidase and nitric oxide synthase [51]. As anti-inflammatory agents, statins cause downregulation of inflammatory cytokines [52] and reduce the concentrations of C-reactive protein (CRP) in hypercholesterolemia [53], as observed in the mice from the HLS group in this study. Kagami et al. (2008) demonstrated that simvastatin decreases the expression of TNFα and IL-6 mRNA in mast cells, thereby attenuating the inflammatory process by reducing IL-1β [54] and the secretion of MCP-1 and MMPs in mice deficient in the Apolipoprotein E (APOE) gene [55] as observed from the expression of MMP-2 and MMP-9 in the mice of the HLS group. Furthermore, statins decrease the expression of CD40 and the activation of vascular cells related to CD40 [56]. However, in the present study, we observed that simvastatin also decreased the expression of CD40L in the myocardium of dyslipidemic mice. Therefore, it can be concluded that statins block the CD40/CD40L pathway in the myocardium, protecting it against oxidative and inflammatory stress.

The prevention of the inflammatory process and oxidative stress observed in the HLU and HLS groups reduced HDL oxidation and its hepatic removal. Thus, we found no reduction in the serum levels of HDLc in the mice of these groups. This is crucial in cardiac protection since, in addition to the reverse cholesterol transport function, it has an antioxidant effect [12] due to its ability to destroy the lipid hydroperoxides that oxidize LDLc phospholipids through the action of paraoxonase-1 and paraoxonase-3 [57]. The HLU and HLS groups showed high levels of HDLc and low serum levels of PCR. In addition to the direct anti-inflammatory effect of the phenolic compounds in grape juice and simvastatin, this increase in HDLc levels also had an anti-inflammatory effect in these animals [58], thus preventing oxidative stress, improving vasorelaxation via nitric oxide, and preventing an increase in blood pressure in the mice of the HLU and HLS groups, which probably reduced the afterload in these mice to prevent LVH.

The HL group showed an increase in serum insulin levels and insulin resistance. Insulin causes tyrosine phosphorylation of the insulin receptor substrate (IRS-1). However, insulin-inducing agents, such as free fatty acids, oxidative stress, and inflammation, activate serine IRS-1 phosphorylation kinases, which inhibited their function [59]. The mice in the HL group showed no hyperglycemia. Insulin resistance in the absence of hyperglycemia found in the HL group in this study may have occurred due to the ability of IRS-2 to partially compensate for the absence of IRS-1 [60].

The HLU and HLS groups displayed high HDLc levels and did not show an increase in insulin levels and insulin resistance compared to the HL group. In addition to the antioxidant action of HDLc molecules in the HLU group, phenolic compounds, especially the anthocyanins present in grape juice, may also have played an important antioxidant role, since anthocyanins can attenuate oxidative stress. This can occur due to the inhibition of LDLc oxidation due to its chemical structure, such as the degree of glycosylation and the number of hydroxyl groups [61]. The mice in the HLU and HLS groups did not exhibit insulin resistance associated with hyperinsulinemia, suggesting that the antioxidant/anti-inflammatory effect of HDLc and phenolic compounds present in grape juice prevented the oxidation of insulin receptor substrates.

In conclusion, grape juice (*Vitis labrusca* L.) possesses potential hypolipidemic and cardiac protective activities similar to those of simvastatin, which prevent insulin resistance by a direct antioxidant action of phenolic compounds, or indirectly by antioxidant and anti-inflammatory activities of HDLc, thus demonstrating that grape juice is a functional food having a great potential to prevent cardiovascular diseases.

## Supporting information

S1 File (PDF)
