# Supplementary material for: Grape juice attenuates left ventricular hypertrophy in dyslipidemic mice
Source: PLoS One. 2020 Sep 3;15(9):e0238163. doi: 10.1371/journal.pone.0238163 (PMC7470265; doi:10.1371/journal.pone.0238163)
Supplement: S1 File — (PDF) [file pone.0238163.s001.pdf]

COMITÊ DE ÉTICA NO USO DE ANIMAIS – CEUA/IFSULDEMINAS

Parecer N°: 03/A 2014.

A Comissão de Ética no Uso de Animais (CEUA), do Instituto Federal do Sul de Minas Gerais, após analisar o projeto de pesquisa intitulado: Influência de fitoterápicos e alimentos funcionais na dislipidemia, na hipertrofia ventricular esquerda e na aterosclerose de camundongos dislipidêmicos, do pesquisador Professor Dr. José Antonio Dias Garcia, decidiu enquadrá-lo na categoria de **APROVADO**.

Machado, 30 de Setembro de 2014.

Professor Dr. José Antônio Dias Garcia
Coordenador do CEUA – IFSULDEMINAS

Apresentação de relatório Final – 30/09/2016

Modelo de relatório = NIPE.
